# Inflammatory cytokines and growth factors were not associated with psychosis liability or childhood trauma

**DOI:** 10.1371/journal.pone.0219139

**Published:** 2019-07-05

**Authors:** Jacqueline Counotte, Veerle Bergink, Roos Pot-Kolder, Hemmo A. Drexhage, Hans W. Hoek, Wim Veling

**Affiliations:** 1 Parnassia Psychiatric Institute, The Hague, The Netherlands; 2 Department of Psychiatry, Erasmus Medical Center, Rotterdam, The Netherlands; 3 Department of Clinical Psychology, VU University, Amsterdam, The Netherlands; 4 Department of Immunology, Erasmus Medical Center, Rotterdam, The Netherlands; 5 Department of Psychiatry, University Medical Center Groningen, University of Groningen, Groningen, The Netherlands; 6 Department of Epidemiology, Mailman School of Public Health, Columbia University, New York, NY, United States of America; Chiba Daigaku, JAPAN

## Abstract

Psychosis is a multifactorial condition arising from an interaction between genetic liability and exposure to environmental risk factors, in particular childhood trauma. Furthermore, accumulating evidence supports a role for the immune system in the aetiology of psychosis. Increased peripheral levels of pro-inflammatory cytokines and reduced neurotrophic factors are found in patients with psychosis. Childhood trauma is highly prevalent in psychosis patients and is also associated with increased pro-inflammatory cytokines and reduced neurotrophic factors. Recent studies suggest the increase in pro-inflammatory cytokines and decrease in neurotrophic factors seen in psychosis may be attributable to the effects of child maltreatment. The aim of this study was to improve understanding of the relation between childhood trauma, inflammation and psychosis. We examined separate and interaction effects of psychosis liability and childhood trauma on serum levels of BDNF, CCL-2, CRP, IFN-γ, IGFBP2, IL-6, PDGF, SCF and TNF-α in 40 patients with recent onset psychosis, 13 patients at Ultra-High Risk (UHR) for psychosis, 31 unaffected siblings of psychosis patients and 41 healthy controls. Childhood trauma was assessed retrospectively with the Childhood Trauma Questionnaire (CTQ). No statistically significant effects of psychosis liability or childhood trauma on concentrations of cytokines or growth factors in peripheral blood were found, nor were there any statistically significant interaction effects of psychosis liability with childhood trauma on serum levels of cytokines and growth factors.

## Introduction

Psychosis is a multifactorial condition arising from an interaction between genetic liability and exposure to environmental risk factors[1]. The traditional biological explanatory model includes neurodevelopmental abnormalities and dopamine dysregulation. In recent decades, this view has been expanded by a hypothesized role for the immune system in the aetiology of psychosis. Counts and function of immune cells, including monocytes and T cells and serum levels of cytokines were shown to be altered in the peripheral blood of psychosis patients [2]. While interleukin (IL)-12, interferon (IFN)-y, tumor necrosis factor (TNF)-α and soluble IL-2 receptor (sIL-2s) are elevated in both recent onset as well as chronic and medicated psychosis patients, pro-inflammatory cytokines IL-1β, IL-6 and transforming growth factor (TGF)-β are elevated only in patients with recent onset psychosis and acute relapse and normalized after antipsychotic treatment [3]. A recent study on a large series of 180 antipsychotic-naive schizophrenia patients from four different sites [4] confirmed abnormal levels of both pro- and anti-inflammatory cytokines, which were also dependent on the duration and treatment of the disease. Immune dysregulation can affect brain function via several pathways [5,6]. For example, some cytokines can pass the blood brain barrier and are neurotoxic or induce neuroinflammation [7]. Also, the immune system has a beneficial role in neurodevelopment and plasticity which can be disturbed by both deficient and exaggerated immune activation [8]. Furthermore, the immune system is connected to other regulatory systems, e.g. the neuroendocrine stress response, glutamate transmission and secretion of neurotrophic factors. Inflammation has been linked to decreased brain-derived neurotrophic factor (BDNF) levels [9]. BDNF is a growth factor essential for neurodevelopment and plasticity. Reduced BDNF levels have been reported in schizophrenia [10]. It is still largely unknown whether altered levels of cytokines and (neuro)trophic factors in psychosis are a cause or consequence of psychotic diseases or an intermediate factor linking other risk factors to psychosis.

Childhood trauma was consistently shown to be associated with psychotic disorders in a dose-response manner, across varying designs including large prospective studies [11]. Psychosocial stress activates the hypothalamic-pituitary-adrenal axis, resulting in release of glucocorticoids. Glucocorticoid receptors are ubiquitously expressed in immune cells. The acute effects of glucocorticoids are largely immunosuppressive [12,13]. However, prolonged stress especially during critical time frames may affect the immune system differentially and result in low grade inflammation [14]. Childhood maltreatment during the first decade of life was associated with markers of inflammation in adults [15]. A dose-response relation was found between severity of maltreatment and increased levels of high sensitive C-reactive protein (CRP), fibrinogen and white blood cell count. In a population of medicated and chronic patients with schizophrenia or schizoaffective disorder, IL-6 and TNF-α were significantly raised in the subset of 24 out of 40 patients that reported a history of childhood trauma compared to healthy and non-traumatized controls [16]. In the group of patients that did not report childhood trauma, levels of pro-inflammatory cytokines were comparable to healthy controls. In patients with recent onset psychosis, hsCRP levels were raised only in the subgroup of patients who had experienced sexual abuse [17].

These studies suggest the increase in pro-inflammatory cytokines and decrease in neurotrophic factors seen in psychosis may be attributable to the effects of child maltreatment. Another possibility is that individuals at (genetic) risk for psychosis are more vulnerable to effects of child maltreatment on the immune system. It is still largely unknown how psychosis liability and childhood trauma interact. Furthermore, only healthy controls and psychosis patients, but not other psychosis liability groups have been examined.

The aim of this study was to improve understanding of the association between childhood trauma, inflammation and psychosis. It concerns a secondary analysis of a previously described study sample [18]. We examined serum levels of cytokines and growth factors, namely BDNF, chemokine (C-C motif) ligand (CCL)-2, CRP, IFN-γ, insulin-like growth factor binding protein (IGFBP2), IL-6, platelet-derived growth factor (PDGF), stem cell factor (SCF) and TNF-α in groups with different liability for psychosis, with and without the experiences of childhood trauma.

Psychosis can be seen as the expression of a psychosis phenotype along a continuum [19,20], ranging from individuals with increased risk to psychotic disorder, to individuals with mild or nonspecific symptoms, to individuals with moderate, subthreshold symptoms and functional decline, to people who have a single psychotic episode and finally severe and unremitting psychotic illness [19]. In this study, four groups along this psychosis continuum were selected: patients with recent-onset psychotic disorder, patients with moderate, subthreshold psychotic symptoms (so-called ultra-high risk for psychosis), unaffected siblings of patients with psychotic disorder and healthy controls. Furthermore, (interaction) effects of lower vs. higher psychosis liability and childhood trauma on serum levels of cytokines and growth factors were examined.

## Methods

### Participants

This study was a secondary analysis in a subset of a previously described study sample [18]. Individuals aged 18–35 with different phenotypic liability to psychosis were included: (1.) 40 patients with a first diagnosis of any psychotic disorder—except for substance-induced psychotic disorder and psychotic disorder due to a medical condition—established within the last five years and (2.) 13 Patients at Ultra-High Risk (UHR) for psychosis (3.) 31 unaffected siblings of patients diagnosed with a psychotic disorder and (4.) 41 healthy controls. Patients and siblings were recruited from psychiatric institutions in The Hague, Rotterdam, Delft and Castricum in the Netherlands. UHR patients were recruited from the patient population of the Early Detection and Intervention Team (EDIT) implemented in these institutions. Details of the screening methodology of EDIT are described elsewhere [21]. Briefly, all referrals to secondary mental healthcare facilities were pre-screened using the self-report Prodromal Questionnaire [22]. Those scoring above the cut-off score for subclinical positive psychosis symptoms were further assessed in a semi-structured clinical interview using the Comprehensive Assessment of At-Risk Mental States (CAARMS) [23] to determine presence, severity, frequency and distress of UHR symptoms. Criteria for UHR are based on the positive symptoms subscale (including unusual thought content, non-bizarre ideas, perceptual abnormalities and disorganized speech). The UHR patients included in this study all had both subclinical psychotic symptoms as determined by the CAARMS as well as a decline in social functioning as determined by the Social and Occupational Functioning Assessment Scale (SOFAS) [24].

Controls were recruited from the same communities through flyers in public facilities, including schools for vocational education and dentistry practices. Exclusion criteria were poor command of the Dutch language, an IQ lower than 75 and a history of epilepsy or auto-immune disorder. Psychosis patients and UHR patients were classified as high psychosis liability and siblings and healthy controls were classified as low psychosis liability, based on (1.) phenotype of (subsyndromal) psychotic symptoms which was present in UHR and psychosis patients and absent in siblings and healthy controls and (2.) life time risk for psychosis, which is 100% in psychosis patients, 36% in UHR patients [25], 10% in siblings and 3% in controls from the general population [26]. The study was approved by the medical ethical committee of Leiden University Medical Centre. Participants received verbal and written information about the study and were given the opportunity to ask questions to the study or an independent researcher. At the start of experiments researchers repeated their explanation and made sure the participants understood all procedures, before written informed consent was obtained from all participants.

### Questionnaires

Electronic self-report questionnaires were administrated to obtain information about medical history, length, weight, use of psychotropic and other (including over-the-counter) medication, substance use (smoking, alcohol, cannabis/THC and illicit drugs) and sociodemographic characteristics including sex, age, ethnicity and education level. Childhood trauma was assessed retrospectively with the Childhood Trauma Questionnaire Short Form (CTQ-SF), a well validated 25-item self-report questionnaire including five subscales: emotional abuse, emotional neglect, physical abuse, physical neglect and sexual abuse[27]. Childhood trauma was defined as present if any subscale scores was classified as moderate or severe according to published norm scores [28].

### Serum measures

Blood samples were taken before questionnaires and collected in clotting tubes (5 ml) for serum preparation. Serum samples were stored in liquid nitrogen to enable testing patient and control immune cells in the same experiment. IFN-y and TNF-α were measured using high sensitive ELISA kits (eBioscience) according to the manufacturer’s instructions. Briefly, stock sera were defrosted at 4 ºC. Pre-manufactured microwells absorbed with IFN-γ coating antibody or TNF-α coating antibody were incubated with 50 μl of sample (diluted 2-fold) and 50 μl Biotin-conjugated anti-human TNF-α antibody or anti-human IFN-γ antibody at room temperature on a microplate shaker. Microwells were washed and subsequently incubated with Streptavidin-HRP. After washing, amplification agent 1 (Biotinyl-Tyramide) was added and washed away after 15 minutes. Amplification reagent II (streptavidin-HRP) was added and washed away after 30 minutes. Finally, the wells were incubated with substrate solution and the reaction was terminated by addition of 1 M Posphoric acid. Absorbance was measured at 450 nm as the primary wave length and 620 nm as reference wave length. Each plate contained samples from all liability groups, a low and a high control and 7 human standard dilutions, all performed in duplicate. A standard curve was prepared using a 5-parameter curve fit. For IFN-y, 16 samples above detection limit were diluted 1:6 and repeated.

A premixed multi analyte Luminex kit (LXSAHM, R&D) was used to measure BDNF, CCL-2, PDGF-BB and SCF. In short, 50 μl of sample (diluted 2-fold) or standard was incubated with microparticle cocktail (color-coded magnetic microparticles pre-coated with analyte specific antibodies) at room temperature on a microplate shaker. Any unbound substances were washed away using a magnetic plate washer (BioPlex, Bio-Rad). Microwells were subsequently incubated with a biotinylated antibody cocktail specific to the analytes, washed and incubated with streptavidin-phycoerythrin conjugate. After a final wash, the microparticles were resuspended in buffer and read on a Luminex MAGPIX Analyzer (Bio-Rad). Each plate contained one sample from participants from all liability groups and 6 standard dilutions performed in duplicate.

IGFBP-2 was measured using an ELISA-quantikine (R&D) according to the manufacturer’s instructions. Sample and standard dilutions were assayed in duplicate. Participants with CRP levels above 10 ug/ml and were excluded from analysis as this may be indicative of acute infections. This was the case for eight participants (2 healthy controls, 2 siblings, 2 UHR patients and 2 psychosis patients).

Range and sensitivity of assays is shown in Table 1. The lowest point of each calibration curve was considered the lower limit of quantification (LLOQ) and the highest point of each calibration curve the upper limit of quantification (ULOQ). Number of values outside limits of quantification are shown in Table 1. Where extrapolated values were available, these were used. Values below the lower limit of detection were imputed with values representing 0.5 times the lowest (extrapolated) value. Values above the upper limit of detection were imputed with value 1.5x the highest (extrapolated) value.

**Table 1:** Assay sensitivity.

	BDNF	CCL2	CRP	IFN-y	IGFBP-2	IL-6	PDGF	SCF	TNF-α
Technique	Luminex	Luminex	hsELISA	hsELISA	ELISA	hsELISA	Luminex	Luminex	hsELISA
Range of quantification	36.4–26500 pg/ml	65.34–15880 pg/ml	0.4–10 μg/ml	0.31–20 pg/ml	15.5–1000 ng/ml	0.78–5 pg/ml	44.04–10700 pg/ml	102.88–25000 pg/ml	0.62–40 pg/ml
N	116	115	117	117	114	117	115	115	117
Within limits of detection N (%)	116 (100)	115 (100)	115 (98.2)	84 (71.8)	114 (100)	115 (98.2)	111 (96.5)	115 (100)	83 (70.9)
Below LLOD (N)	0	0	2	28	0	0	0	0	34
Above ULOD (N)	0	0	n.a.	5	0	2	4	0	0
Within limits of quantification N (%)	108 (93.1)	115 (100)	90 (76.9)	56 (47.8)	114 (100)	54 (46.2)	86 (74.8)	36 (31.3)	29 (24.8)
Below LLOQ (N)	0	0	25	22	0	54	0	79	54
Above ULOQ (N)	8	0	n.a.	6	0	5	25	0	0

LLOD = lower limit of detection, ULOD = upper limit of detection LLOQ = lower limit of quantification, ULOQ = upper limit of quantification. Participants with CRP levels above 10 ug/ml were excluded from analysis.

For three samples (1 healthy control and 2 psychosis patients), there was insufficient material to perform all assays.

### Statistics

All analyses were conducted with IBM SPSS version 23. Significance was assumed at *α* < 0.05 (two-tailed). Continuous variables were inspected for normal distribution and log transformed (ln) to achieve a normal distribution if necessary. Sociodemographic characteristics and serum factor levels of cytokines and growth factors were compared between the four groups using one-way analysis of variance (ANOVA) with three predetermined contrasts (high vs. low liability, psychosis vs. UHR and siblings vs. controls) and explorative post-hoc Dunnett’s t-tests for continuous variables and χ^2^ tests for categorical variables.

We used linear or logistic regression models to examine (interaction) effects of psychosis liability and childhood trauma. We explored association of covariates (age, sex, ln(BMI), smoking, cannabis/THC use, ethnicity, education and use of contraceptive or other relevant medication) with outcome measures by entering them separately in MANOVAs. Covariates with associations with outcome measures at significant or trend level (p < 0.1) were selected for correction of regression models. The basic model included only covariates. Subsequently, psychosis liability was added. Next, childhood trauma was added. Lastly, the interaction term psychosis liability x childhood trauma was added. For significant results, we subsequently added psychotropic medication.

## Results

### Sociodemographic characteristics

After exclusion of participants with CRP values above 10 μg/ml, data was available for 39 healthy controls, 29 siblings, 11 ultra-high risk (UHR) and 38 psychosis patients. Sociodemographic characteristics are shown in Table 2. Groups differed significantly on gender, smoking, education level and use of psychotropic medication. Psychosis patients were significantly more likely to be male (χ^2^ (1) = 12.24, p <0.001) than healthy controls. UHR patients were significantly more likely to smoke than healthy controls (χ^2^ (1) = 11.07, p = 0.001) and there was a trend for psychosis patients to smoke more often than healthy controls (χ^2^ (1) = 3.649, p = 0.056). Both UHR and psychosis patients had on average finished lower levels of education than healthy controls (τ_B_ = -2.77, p = 0.048 and τ_B_ = -0.286, p = 0.004 for UHR and psychosis patients, respectively). As expected, both UHR and psychosis patients were also significantly more likely to use psychotropic medication compared to healthy controls (χ^2^ (1) = 28.86, p <0.001 and χ^2^ (1) = 40.29, p <0.001 for UHR and psychosis patients, respectively). For none of the UHR patients this included an antipsychotic. The sample included 12 psychosis patients that did not use psychotropic medication.

**Table 2:** Sociodemographic characteristics.

	Low psychosis liability		High psychosis liability		
	Controls	Siblings	UHR	Psychosis	
	N = 39	N = 29	N = 11	N = 38	*p*
Male	18 (46.2)	17 (58.6)	5 (45.5)	32 (84.2)	0.004
Age	24.0 (21.0–26.0)	25.5 (21.3–30.0)	24.0 (20.0–29.0)	25.5 (23.0–30.0)	0.438
BMI	22.8 (20.2–24.6)	23.6 (21.0–24.8)	23.1 (18.5–26.2)	23.0 (20.4–24.3)	0.979
Native Dutch	29 (74.4)	19 (67.9)	8 (72.7)	21 (55.3)	0.328
Education					
No/primary	0 (0)	0 (0)	0 (0)	3 (7.9)	0.002
Vocational	10 (25.6)	9 (32.1)	7 (63.6)	17 (44.7)	
Secondary	8 (20.5)	2 (7.1)	1 (9.1)	7 (18.4)	
Higher	21 (53.8)	17 (60.7)	3 (27.3)	11 (28.9)	
Smoking	6 (23.1)	7 (29.2)	9 (81.8)	16 (47.1)	0.005
Cannabis use	8 (20.5)	2 (7.1)	4 (36.4)	10 (26.3)	0.138
Medication					
Psychotropic	0 (0)	1 (3.6)	7 (63.6)	26 (68.4)	<0.001
Contraceptive^a^	12 (57.1)	3 (27.3)	4 (66.7)	1 (16.7)	
Other	3 (7.7)	1 (3.6)	1 (9.1)	5 (13.2)	0.584

Values displayed are median (interquartile range) or N (%). P-values of ANOVA (for continuous variables), X^2^ tests (for dichotomous variables) or Kendall’s tau-B (for education) are given. BMI = body mass index. Smoking during last 24 hours. Cannabis use during last month.

^a^ Percentage of all females within group.

### Serum measures

**Fig 1:**
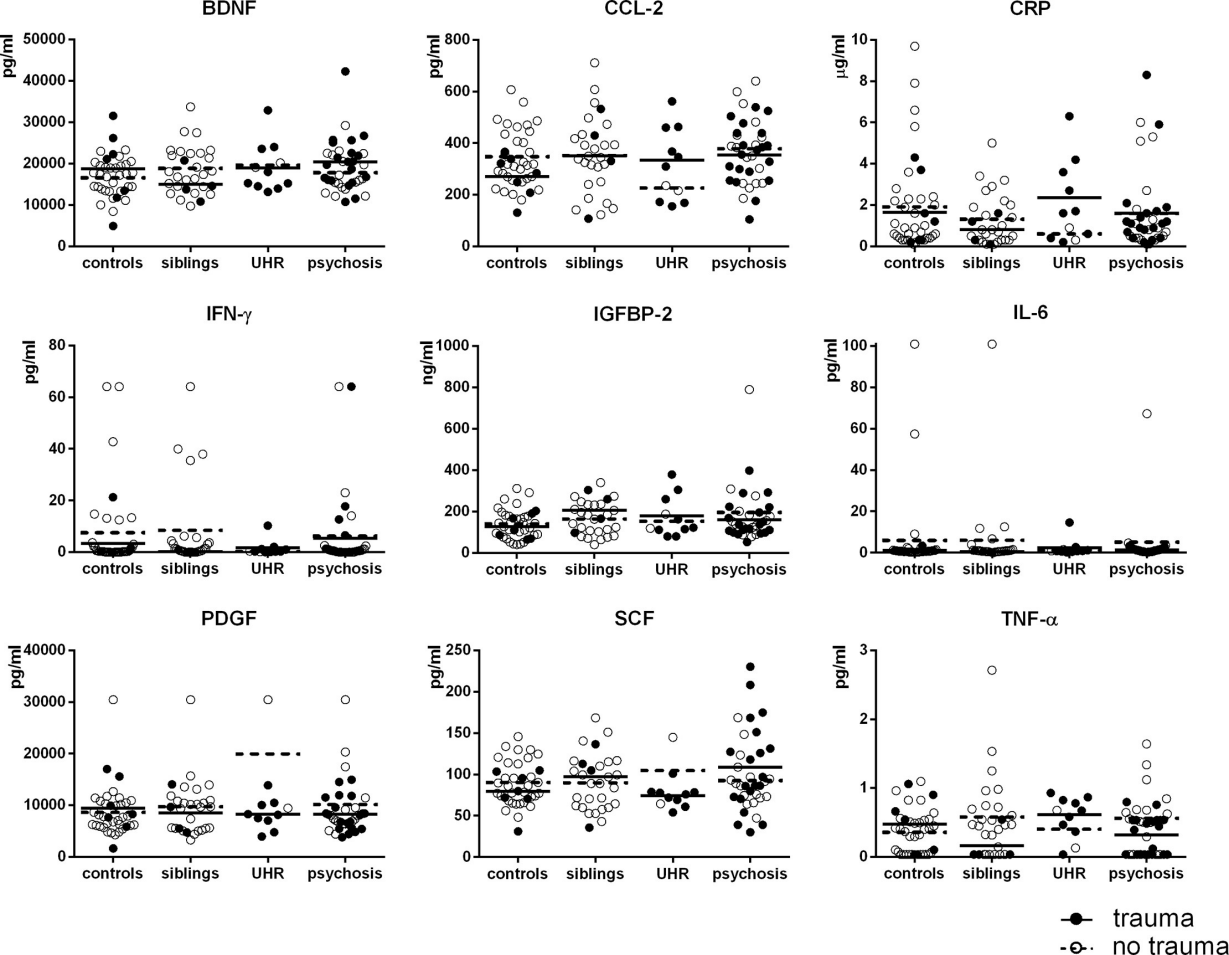
Serum levels of cytokines and growth factors in healthy controls, siblings, UHR and psychosis patients. Out of range concentrations on the lower or upper end of detection were imputed with values representing 0.5 times the lowest value or 1.5 times the highest value, respectively.

Serum levels of BDNF, CCL-2, CRP, IFN-γ, IGFBP-2, IL-6, PDGF, SCF, TNF-α in controls, siblings, UHR and psychosis patients with and without childhood trauma are depicted in Fig 1. Differences in serum levels between controls, siblings, UHR and psychosis patients were explored with uncorrected one way analysis of variance (ANOVA). No significant group differences were found in serum levels of BDNF, CCL-2, CRP, IFN-γ, IGFBP-2, IL-6, PDGF, SCF, TNF-α (Table 3). Results of predetermined contrasts (high vs. low liability, psychosis vs. UHR and siblings vs. controls) and explorative post-hoc Dunnett’s t-tests are available in supplemental data.

**Table 3:** ANOVA test statistics.

	*F*	*df*	*p*	η^2^
Ln(BDNF)	1.296	3, 112	0.279	0.034
Ln(CCL-2)	0.652	3, 111	0.583	0.017
Ln(CRP)	0.673	3, 113	0.570	0.018
Ln(IFN-γ)	0.318	3, 113	0.813	0.008
Ln(IGFBP-2)	1.435	3, 110	0.237	0.038
Ln(IL-6)	0.290	3, 113	0.832	0.008
Ln(PDGF)	0.264	3, 111	0.851	0.007
Ln(SCF)	0.690	3, 111	0.560	0.018
Ln(TNF-α)	0.924	3, 113	0.432	0.024

Four psychosis liability groups (healthy controls, unaffected siblings, UHR patients and psychosis patients) were tested using one-way analysis of variance. *η*^2^ = partial eta squared effect size (0.01–0.06: small effect, 0.06–0.014: moderate effect > 0.14: large effect).

### Childhood trauma

**Fig 2:**
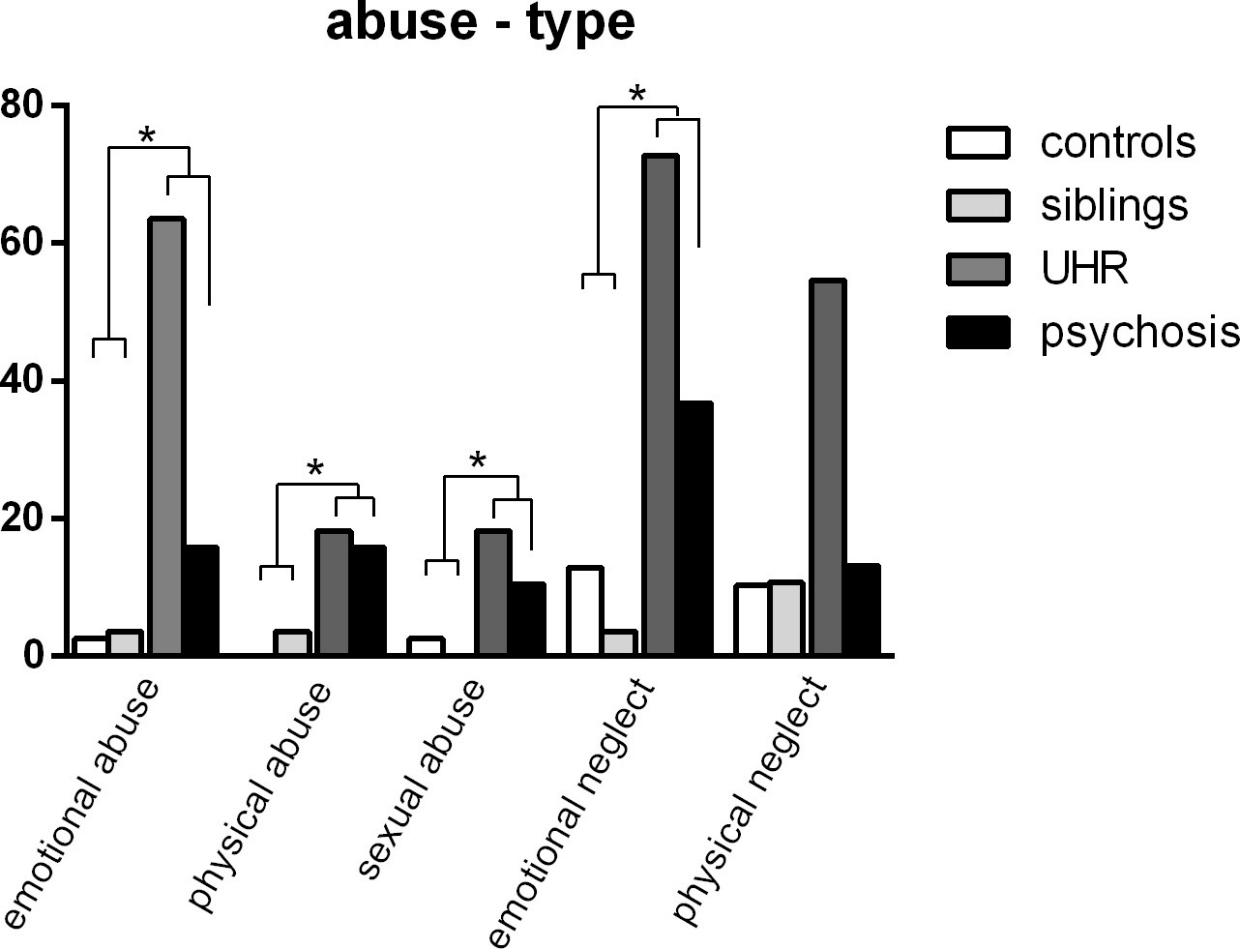
Childhood trauma as reported by groups with different psychosis liability. The Childhood Trauma Questionnaire (CTQ) was used to assess different types of abuse and neglect retrospectively. Percentages of participants within each group that reported moderate or severe levels of emotional abuse, physical abuse, sexual abuse, emotional neglect and physical neglect are shown.

Childhood trauma was reported by 17.9% of controls, 13.8% of siblings, 81.8% of UHR and 52.6% of psychosis patients. Both UHR and psychosis patients reported childhood trauma significantly more often than healthy controls (χ^2^ (1) = 16.09, p <0.001 and χ^2^ (1) = 10.17, p = 0.001 for UHR and psychosis patients, respectively). Type and number of traumas are depicted in Fig 2. All types of trauma were more likely to be reported by high liability group (UHR and psychosis patients) compared to the low liability group (healthy controls and siblings). These differences were significant for emotional abuse (χ^2^ (1) = 13.94, p < 0.001), physical abuse (χ^2^ (1) = 8.70, p = 0.003), sexual abuse (χ^2^ (1) = 5.77, p = 0.016) and emotional neglect (χ^2^ (1) = 19.97, p < 0.001) and at trend level for physical neglect (χ^2^ (1) = 3.11, p = 0.078).

### Psychosis liability x childhood trauma

Separate and interaction effects of psychosis liability and childhood trauma on serum levels were examined using linear regression models. Sex, age, BMI, smoking, cannabis use, education and oral contraceptive use were selected as covariates, as they were associated with outcome measures in MANOVA significantly or at trend level (*p* < 0.10). Regression coefficients of corrected models are shown in Table 4. Regression coefficients of uncorrected models and models with additional correction for psychotropic use are available in supplemental data. Addition of group, childhood trauma or the interaction term did not significantly improve the model for any of the cytokines and growth factors. There were no significant or trend level main or interaction effects of psychosis liability or trauma on growth factors and cytokines.

**Table 4:** Regression coefficients.

	Psychosis liability				Childhood trauma				Psychosis liability x childhood trauma			
	B	[CI]		p	B	[CI]		p	B	[CI]		p
BDNF	-0.02	[-0.18;	0.15]	0.837	-0.02	[-0.23;	0.19]	0.875	0.02	[-0.24;	0.29]	0.860
CCL-2	-0.17	[-0.41;	0.08]	0.179	0.00	[-0.31;	0.31]	0.979	0.08	[-0.32;	0.48]	0.689
CRP	0.07	[-0.56;	0.71]	0.819	0.02	[-0.81;	0.85]	0.960	0.11	[-0.94;	1.16]	0.838
IFN-γ	0.14	[-0.11;	0.40]	0.258	0.20	[-0.11;	0.51]	0.195	-0.28	[-0.68;	0.12]	0.163
IGFBP-2	0.47	[-0.21;	1.16]	0.172	-0.23	[-1.12;	0.66]	0.603	-0.38	[-1.51;	0.74]	0.500
IL-6	0.02	[-1.40;	1.44]	0.981	-1.14	[-2.99;	0.71]	0.224	1.16	[-1.18;	3.51]	0.327
PDGF	0.07	[-0.18;	0.32]	0.587	0.20	[-0.12;	0.52]	0.218	-0.37	[-0.78;	0.04]	0.073
SCF	-0.11	[-0.34;	0.11]	0.321	0.09	[-0.20;	0.37]	0.540	-0.01	[-0.38;	0.35]	0.943
TNF-α	0.39	[-0.45;	1.23]	0.360	-0.75	[-1.84;	0.35]	0.179	0.73	[-0.66;	2.12]	0.297

Regression coefficients of linear regression models are given. Models included psychosis liability (high vs. low), childhood trauma (yes/no) and psychosis liability x childhood trauma as predictors and were corrected for sex, age, BMI, smoking, cannabis use, education and oral contraceptive use.

## Discussion

We examined separate and interaction effects of psychosis liability and childhood trauma on serum levels of cytokines and growth factors. No statistically significant effects of psychosis liability or childhood trauma on concentrations of cytokines or growth factors in peripheral blood were found, nor were there any statistically significant interaction effects of psychosis liability with childhood trauma on serum levels of cytokines and growth factors.

It is possible that the sample size in our study was too small to reach statistically significance. Furthermore, selection bias cannot be ruled and could have resulted in a relatively healthy patient population. The disease trajectories of the recent onset psychosis patients in this sample are still unknown, some will completely recover and not experience any other psychotic episodes, whereas others will develop a more chronic debilitating pattern of disease. A large proportion was taking antipsychotic medication and many may have been (partly) in remission. Similarly, a large portion of the UHR sample will develop a full-blown psychotic episode, whereas in others, the psychotic-like symptoms will lessen and/or functioning will improve. Thus, homeostasis may still be maintained or already restored in our sample.

Confounding factors may also explain differences between our negative results and previous findings. BMI, sex and age are known to affect cytokine levels in peripheral blood [29–31] and were indeed associated with outcome measures in our sample. Especially obesity is considered a likely confounder in psychosis studies, as it is linked with inflammatory markers, more common among psychosis patients and further increased by the use of psychotropic medication use [32,33]. We corrected for BMI, sex and age in our sample. Furthermore, in contrast to many previous studies, our sample was characterized by an on average healthy BMI, with no statistically significant differences in BMI between groups. In the sample of Dennison et al., patients were considerable older, more likely to be obese and all used psychotropic medication, often a combination of multiple agents [16]. Hepgul et al. studied first-episode psychosis patients and found CRP levels to be increased in the subset of patients who were traumatized, specifically by the experience of sexual abuse [17]. Even these first-episode psychosis patients had significantly higher BMI than controls. Moreover, BMI was associated with both sexual abuse and CRP levels and thus could have confounded results. In a recent study examining the influence of BMI on cytokines in drug naive first-episode psychosis patients, IL-8 was the only cytokine increased in normal-weight patients compared to normal-weight controls [34], whereas over-weight patients had increased levels of IL-6, IL-8, CCL-4, IL-13, IL-2, IL-7, IL-12p70 and IL-23 compared to over-weight controls [34]. Interestingly, there was no difference between over- and normal-weight controls, whereas overweight patients had increased concentrations of inflammatory markers compared to normal-weight patients. While no formal interaction test was performed in this study, these findings are suggestive of an interaction effect between obesity and psychosis liability. Such an interaction effect would explain the negative results of our study where psychosis liability was present, but obesity relatively rare. Thus, obesity may be an essential player in the complex relation between psychosis, metabolic syndrome and immune dysregulation rather than a simple confounding factor.

Patients with psychotic disorders, including this study sample, form a heterogenous group and previous work has shown the course of immune dysregulation in psychosis patients to be highly dynamic, involving both pro- and anti-inflammatory forces. Cytokines and growth factors in peripheral blood are the end-product of diverse processes and players, including non-immune cells, e.g. adipocytes and endothelial cells. Immune dysregulation may occur without affecting serum cytokine levels, especially as pro-inflammatory mechanisms may be—temporarily—compensated by an increased anti-inflammatory response.

### Limitations

Childhood trauma was measured retrospectively, which has the potential for recall bias. We used the Childhood Trauma Questionnaire, which is considered a well-validated and reliable instrument [27,35]. Furthermore, retrospective report of childhood trauma by psychosis patients was shown to be reliable [36]. We previously reported on the increased prevalence of childhood trauma in patients with high psychosis liability in this sample and found that patients with a history of childhood trauma reported more psychotic and affective symptoms in daily life and more paranoid ideation and stress after exposure to social stress in a virtual reality environment [37]. Our findings are in line with previous research, finding a high prevalence of childhood trauma in UHR [38] and psychosis patients [11] and increased prevalence of childhood trauma in psychosis patients compared to unaffected siblings [39]. In this secondary analysis, we confirmed increased prevalence of childhood trauma in UHR and psychosis patients in this subset and show that all subtypes of traumas had an increased prevalence, with a trend level effect for physical abuse and statistically significant effects for all other subtypes.

We selected four group along the psychosis liability spectrum. While psychosis liability is a continuous concept, we dichotomized it into lower and higher liability to analyse (interaction) effects of childhood trauma. The sample size was too small to analyse groups separately, especially as childhood trauma was extremely common among UHR, limiting the number of UHR patients without childhood trauma. We considered classifying siblings and UHR patients as an intermediate risk group, but as siblings were more comparable to controls both in terms of life time psychosis risk and phenotype, we considered it more valid to classify controls and siblings as lower and UHR and psychosis patients as higher psychosis liability groups. The life time risk of psychotic disorders is approximately 3% for controls (general population), 10% for siblings[26], 36% for UHR [25] and, by definition, 100% for patients with recent onset psychotic disorder. UHR and psychosis patients all reported (subsyndromal) psychotic symptoms, which were uncommon among siblings and controls from the general population.

We used high sensitive assays. Still, some values were outside the limit of detection, either being undetectably low or out of range high. Furthermore, a larger proportion of values were extrapolated by software as they were outside the calibration curve. The latter proportions were especially high for IFN-γ, IL-6, SCF and TNF-α (Table 1). The reliability of these measurements may be decreased by increased measurement error. However, they are not invalid measurements and still confer meaning and we therefore included them in our analysis. We aimed to be detailed and transparent regarding assay sensitivity and data reliability, but were unable to compare the sensitivity of our assays to other studies as the information reported was very limited.

### Conclusions

We did not find evidence for independent or interaction effect of psychosis liability or childhood trauma on peripheral levels of cytokines and growth factors in this sample. These negative results should be interpreted within a framework of meta-analytic work showing immune deregulation in psychosis—which is known to be highly dynamic, the heterogeneity of patients with psychotic disorders and signs for a complex interaction with obesity.

## Supporting information

S1 Table Results of predetermined contrasts (high vs. low liability, psychosis vs. UHR and siblings vs. controls) and explorative post-hoc Dunnett’s t-tests op one-way analysis of variance of serum levels in four psychosis liability groups: healthy controls, siblings, ultra-high risk (UHR) patients and psychosis patients. Not that p-values for Dunnett’s t-tests are corrected for multiple testing whereas p-values for predetermined contrast t-tests are not.
(DOCX)

S2 Table Uncorrected models. Regression coefficients of uncorrected linear regression models are given. Models included psychosis liability (high vs. low), childhood trauma (yes/no) and psychosis liability x childhood trauma as predictors.
(DOCX)

S3 Table Correction for psychotropic medication use. Regression coefficients of linear regression models are given. Models included psychosis liability (high vs. low), childhood trauma (yes/no) and psychosis liability x childhood trauma as predictors and were corrected for sex, age, BMI, smoking, cannabis use, education, oral contraceptive use and psychotropic medication use.
(DOCX)

## References

[1] van Os J, Kenis G, Rutten BPF. The environment and schizophrenia. Nature. 2010/11/12. 2010;468: 203–12. 10.1038/nature09563 21068828

[2] Bergink V, Gibney SM, Drexhage HA. Autoimmunity, inflammation, and psychosis: a search for peripheral markers. Biol Psychiatry. 2014;75: 324–31. 10.1016/j.biopsych.2013.09.037 24286760

[3] Miller BJ, Buckley P, Seabolt W, Mellor A, Kirkpatrick B. Meta-analysis of cytokine alterations in schizophrenia: clinical status and antipsychotic effects. Biol Psychiatry. 2011;70: 663–71. 10.1016/j.biopsych.2011.04.013 21641581 PMC4071300

[4] De Witte L, Tomasik J, Schwarz E, Guest PC, Rahmoune H, Kahn RS, et al Cytokine alterations in first-episode schizophrenia patients before and after antipsychotic treatment. Schizophr Res. 2014;154: 23–29. 10.1016/j.schres.2014.02.005 24582037

[5] Danese A, Baldwin JR. Hidden Wounds? Inflammatory Links Between Childhood Trauma and Psychopathology. Annu Rev Psychol. 2017;68: 517–544. 10.1146/annurev-psych-010416-044208 27575032

[6] Dantzer R. Neuroimmune Interactions: From the Brain to the Immune System and Vice Versa. Physiol Rev. 2018;98: 477–504. 10.1152/physrev.00039.2016 29351513 PMC5866360

[7] Dantzer R, O’Connor JC, Freund GG, Johnson RW, Kelley KW. From inflammation to sickness and depression: when the immune system subjugates the brain. Nat Rev Neurosci. 2008;9: 46–56. 10.1038/nrn2297 18073775 PMC2919277

[8] Yirmiya R, Goshen I. Immune modulation of learning, memory, neural plasticity and neurogenesis. Brain Behav Immun. 2011;25: 181–213. 10.1016/j.bbi.2010.10.015 20970492

[9] Calabrese F, Rossetti AC, Racagni G, Gass P, Riva MA, Molteni R. Brain-derived neurotrophic factor: a bridge between inflammation and neuroplasticity. Front Cell Neurosci. 2014;8: 1–7. 10.3389/fncel.2014.00001 25565964 PMC4273623

[10] Green MJ, Matheson SL, Shepherd A, Weickert CS, Carr VJ. Brain-derived neurotrophic factor levels in schizophrenia: a systematic review with meta-analysis. Mol Psychiatry. 2011;16: 960–72. 10.1038/mp.2010.88 20733577

[11] Varese F, Smeets F, Drukker M, Lieverse R, Lataster T, Viechtbauer W, et al Childhood adversities increase the risk of psychosis: A meta-analysis of patient-control, prospective-and cross-sectional cohort studies. Schizophr Bull. 2012;38: 661–671. 10.1093/schbul/sbs050 22461484 PMC3406538

[12] Elenkov IJ, Chrousos GP. Stress hormones, proinflammatory and antiinflammatory cytokines, and autoimmunity. Ann N Y Acad Sci. 2002;966: 290–303. Available: http://www.ncbi.nlm.nih.gov/pubmed/12114286 10.1111/j.1749-6632.2002.tb04229.x 12114286

[13] Franchimont D. Overview of the actions of glucocorticoids on the immune response: a good model to characterize new pathways of immunosuppression for new treatment strategies. Ann N Y Acad Sci. 2004;1024: 124–37. 10.1196/annals.1321.009 15265777

[14] Fagundes CP, Glaser R, Kiecolt-Glaser JK. Stressful early life experiences and immune dysregulation across the lifespan. Brain Behav Immun. 2013;27: 8–12. 10.1016/j.bbi.2012.06.014 22771426 PMC3518756

[15] Danese A, Pariante CM, Caspi A, Taylor A, Poulton R. Childhood maltreatment predicts adult inflammation in a life-course study. Proc Natl Acad Sci U S A. 2007;104: 1319–24. 10.1073/pnas.0610362104 17229839 PMC1783123

[16] Dennison U, McKernan D, Cryan J, Dinan T. Schizophrenia patients with a history of childhood trauma have a pro-inflammatory phenotype. Psychol Med. 2012;42: 1865–71. 10.1017/S0033291712000074 22357348

[17] Hepgul N, Pariante CM, Dipasquale S, DiForti M, Taylor H, Marques TR, et al Childhood maltreatment is associated with increased body mass index and increased C-reactive protein levels in first-episode psychosis patients. Psychol Med. 2012;42: 1893–901. 10.1017/S0033291711002947 22260948 PMC4081598

[18] Veling W, Pot-Kolder R, Counotte J, van Os J, van der Gaag M. Environmental Social Stress, Paranoia and Psychosis Liability: A Virtual Reality Study. Schizophr Bull. 2016;42: 1363–1371. 10.1093/schbul/sbw031 27038469 PMC5049523

[19] McGorry PD, Nelson B, Goldstone S, Yung AR. Clinical staging: A heuristic and practical strategy for new research and better health and social outcomes for psychotic and relate mood disorders. Can J Psychiatry. 2010;55: 486–497. 10.1177/070674371005500803 20723276

[20] Van Os J. The dynamics of subthreshold psychopathology: Implications for diagnosis and treatment. Am J Psychiatry. 2013;170: 695–698. 10.1176/appi.ajp.2013.13040474 23820827

[21] Rietdijk J, Dragt S, Klaassen R, Ising H, Nieman D, Wunderink L, et al A single blind randomized controlled trial of cognitive behavioural therapy in a help-seeking population with an At Risk Mental State for psychosis: the Dutch Early Detection and Intervention Evaluation (EDIE-NL) trial. Trials. 2010;11: 30 10.1186/1745-6215-11-30 20307268 PMC2853533

[22] Loewy RL, Bearden CE, Johnson JK, Raine A, Cannon TD. The prodromal questionnaire (PQ): preliminary validation of a self-report screening measure for prodromal and psychotic syndromes. Schizophr Res. 2005;79: 117–25. Available: http://www.ncbi.nlm.nih.gov/pubmed/16276559 16276559

[23] Yung AR, Yuen HP, McGorry PD, Phillips LJ, Kelly D, Dell’Olio M, et al Mapping the onset of psychosis: the Comprehensive Assessment of At-Risk Mental States. Aust N Z J Psychiatry. 2005;39: 964–71. 10.1080/j.1440-1614.2005.01714.x 16343296

[24] Goldman HH, Skodol AE, Lave TR. Revising axis V for DSM-IV: a review of measures of social functioning. Am J Psychiatry. 1992;149: 1148–56. 10.1176/ajp.149.9.1148 1386964

[25] Fusar-Poli P, Borgwardt S, Bechdolf A, Addington J, Riecher-Rössler A, Schultze-Lutter F, et al The psychosis high-risk state: a comprehensive state-of-the-art review. JAMA psychiatry. 2013;70: 107–20. 10.1001/jamapsychiatry.2013.269 23165428 PMC4356506

[26] van Os J, Linscott RJ, Myin-Germeys I, Delespaul P, Krabbendam L. A systematic review and meta-analysis of the psychosis continuum: evidence for a psychosis proneness-persistence-impairment model of psychotic disorder. Psychol Med. 2009;39: 179–95. 10.1017/S0033291708003814 18606047

[27] Bernstein DP, Stein JA, Newcomb MD, Walker E, Pogge D, Ahluvalia T, et al Development and validation of a brief screening version of the Childhood Trauma Questionnaire. Child Abuse Negl. 2003;27: 169–90. Available: http://www.ncbi.nlm.nih.gov/pubmed/12615092 12615092 10.1016/s0145-2134(02)00541-0

[28] Bernstein DP, Fink L. Childhood Trauma Questionnaire: A retrospective self-report. San Antonio, TX: The Psychological Corporation; 1998.

[29] Monteiro R, Azevedo I. Chronic Inflammation in Obesity and the Metabolic Syndrome. Mediators Inflamm. 2010;2010: 1–10. 10.1155/2010/289645 20706689 PMC2913796

[30] Schmidt FM, Weschenfelder J, Sander C, Minkwitz J, Thormann J, Chittka T, et al Inflammatory cytokines in general and central obesity and modulating effects of physical Activity. PLoS One. 2015;10: 1–17. 10.1371/journal.pone.0121971 25781614 PMC4363366

[31] Álvarez-Rodríguez L, López-Hoyos M, Muñoz-Cacho P, Martínez-Taboada VM. Aging is associated with circulating cytokine dysregulation. Cell Immunol. 2012;273: 124–132. 10.1016/j.cellimm.2012.01.001 22316526

[32] Mitchell AJ, Vancampfort D, Sweers K, Van Winkel R, Yu W, De Hert M. Prevalence of metabolic syndrome and metabolic abnormalities in schizophrenia and related disorders-a systematic review and meta-analysis. Schizophr Bull. 2013;39: 306–318. 10.1093/schbul/sbr148 22207632 PMC3576174

[33] Beumer W, Drexhage RCRC, De Wit H, Versnel MA, Drexhage HA, Cohen D, et al Increased level of serum cytokines, chemokines and adipokines in patients with schizophrenia is associated with disease and metabolic syndrome. Psychoneuroendocrinology. 2012;37: 1901–1911. S0306-4530(12)00133-3 [pii] 10.1016/j.psyneuen.2012.04.001 22541717

[34] Juncal-Ruiz M, Riesco-Dávila L, de la Foz VO-G, Ramírez-Bonilla M, Martínez-García O, Irure-Ventura J, et al The effect of excess weight on circulating inflammatory cytokines in drug-naïve first-episode psychosis individuals. J Neuroinflammation. 2018;15: 63 10.1186/s12974-018-1096-6 29490673 PMC6389043

[35] Thombs BD, Bernstein DP, Lobbestael J, Arntz A. A validation study of the Dutch Childhood Trauma Questionnaire-Short Form: factor structure, reliability, and known-groups validity. Child Abuse Negl. 2009;33: 518–23. 10.1016/j.chiabu.2009.03.001 19758699

[36] Fisher HL, Craig TK, Fearon P, Morgan K, Dazzan P, Lappin J, et al Reliability and comparability of psychosis patients’ retrospective reports of childhood abuse. Schizophr Bull. 2011;37: 546–553. 10.1093/schbul/sbp103 19776204 PMC3080697

[37] Veling W, Counotte J, Pot-Kolder R, van Os J, van der Gaag M. Childhood trauma, psychosis liability and social stress reactivity: a virtual reality study. Psychol Med. 2016;46: 3339–3348. 10.1017/S0033291716002208 27619196

[38] Kraan T, Velthorst E, Smit F, de Haan L, van der Gaag M. Trauma and recent life events in individuals at ultra high risk for psychosis: Review and meta-analysis. Schizophr Res. 2015;161: 143–149. 10.1016/j.schres.2014.11.026 25499046

[39] Heins M, Simons C, Lataster T, Pfeifer S, Versmissen D, Lardinois M, et al Childhood trauma and psychosis: A case-control and case-sibling comparison across different levels of genetic liability, psychopathology, and type of trauma. Am J Psychiatry. 2011;168: 1286–1294. 10.1176/appi.ajp.2011.10101531 21955935

