# Supplementary material for: Inflammatory cytokines and growth factors were not associated with psychosis liability or childhood trauma
Source: PLoS One. 2019 Jul 5;14(7):e0219139. doi: 10.1371/journal.pone.0219139 (PMC6611659; doi:10.1371/journal.pone.0219139)
Supplement: S1 Table — Not that p-values for Dunnett’s t-tests are corrected for multiple testing whereas p-values for predetermined contrast t-tests are not. (DOCX) [file pone.0219139.s001.docx]

|  | Post hoc contrast | | | | | | Dunnett’s t test (vs. controls) | | |
| --- | --- | --- | --- | --- | --- | --- | --- | --- | --- |
|  | High vs. low liability | | Siblings vs. controls | | UHR vs. psychosis | | Siblings | UHR | Psychosis |
|  | *t* | *p* | *t* | *p* | *t* | *p* | *p* | *p* | *p* |
| Ln(BDNF) | 1.372 | 0.173 | 1.093 | 0.277 | 0.011 | 0.991 | 0.586 | 0.491 | 0.171 |
| Ln(CCL-2) | -0.114 | 0.909 | 0.065 | 0.948 | 1.298 | 0.197 | 1.000 | 0.843 | 0.722 |
| Ln(CRP) | 0.968 | 0.335 | -1.023 | 0.308 | -0.788 | 0.432 | 0.635 | 0.881 | 0.991 |
| Ln(IFN-γ) | -0.634 | 0.527 | 0.748 | 0.456 | 0.026 | 0.979 | 0.815 | 0.998 | 0.997 |
| Ln(IGFBP-2) | 1.085 | 0.280 | 1.613 | 0.110 | -0.098 | 0.922 | 0.271 | 0.447 | 0.203 |
| Ln(IL-6) | 0.453 | 0.651 | 0.360 | 0.719 | 0.417 | 0.678 | 0.973 | 0.995 | 0.702 |
| Ln(PDGF) | 0.476 | 0.635 | 0.646 | 0.519 | -0.511 | 0.610 | 0.870 | 0.794 | 0.964 |
| Ln(SCF) | -0.086 | 0.932 | 0.063 | 0.950 | 1.324 | 0.188 | 1.000 | 0.845 | 0.692 |
| Ln(TNF-α) | 1.218 | 0.228 | 0.557 | 0.580 | -1.786 | 0.088 | 0.906 | 0.271 | 0.994 |
