# Supplementary material for: Inflammatory cytokines and growth factors were not associated with psychosis liability or childhood trauma
Source: PLoS One. 2019 Jul 5;14(7):e0219139. doi: 10.1371/journal.pone.0219139 (PMC6611659; doi:10.1371/journal.pone.0219139)
Supplement: S2 Table — Regression coefficients of uncorrected linear regression models are given. Models included psychosis liability (high vs. low), childhood trauma (yes/no) and psychosis liability x childhood trauma as predictors. (DOCX) [file pone.0219139.s002.docx]

|  | Psychosis liability | | | |  | Childhood trauma | | | |  | Psychosis liability x childhood trauma | | | |
| --- | --- | --- | --- | --- | --- | --- | --- | --- | --- | --- | --- | --- | --- | --- |
|  | B | [CI] |  | p |  | B | [CI] |  | p |  | B | [CI] |  | p |
| BDNF | 0.03 | [-0.13; | 0.19] | 0.690 |  | -0.08 | [-0.28; | 0.12] | 0.433 |  | 0.16 | [-0.10; | 0.43] | 0.227 |
| CCL-2 | 0.04 | [-0.17; | 0.25] | 0.702 |  | -0.18 | [-0.44; | 0.08] | 0.179 |  | 0.13 | [-0.22; | 0.48] | 0.474 |
| CRP | -0.12 | [-0.72; | 0.48] | 0.693 |  | -0.21 | [-0.96; | 0.55] | 0.590 |  | 0.57 | [-0.44; | 1.58] | 0.267 |
| IFN-γ | 0.20 | [-0.10; | 0.49] | 0.185 |  | 0.05 | [-0.30; | 0.41] | 0.773 |  | -0.14 | [-0.62; | 0.34] | 0.563 |
| IGFBP-2 | 0.25 | [-0.39; | 0.90] | 0.438 |  | -0.59 | [-1.40; | 0.23] | 0.156 |  | 0.30 | [-0.79; | 1.39] | 0.585 |
| IL-6 | -0.39 | [-1.66; | 0.88] | 0.545 |  | -1.42 | [-3.03; | 0.20] | 0.084 |  | 1.19 | [-0.96; | 3.34] | 0.275 |
| PDGF | 0.14 | [-0.11; | 0.39] | 0.259 |  | -0.06 | [-0.37; | 0.25] | 0.698 |  | -0.14 | [-0.56; | 0.27] | 0.501 |
| SCF | 0.04 | [-0.16; | 0.24] | 0.706 |  | -0.08 | [-0.33; | 0.17] | 0.527 |  | 0.06 | [-0.28; | 0.40] | 0.717 |
| TNF-α | 0.22 | [-0.49; | 0.93] | 0.540 |  | -0.48 | [-1.37; | 0.42] | 0.297 |  | 0.21 | [-0.99; | 1.41] | 0.731 |
