# Supplementary material for: Inflammatory cytokines and growth factors were not associated with psychosis liability or childhood trauma
Source: PLoS One. 2019 Jul 5;14(7):e0219139. doi: 10.1371/journal.pone.0219139 (PMC6611659; doi:10.1371/journal.pone.0219139)
Supplement: S3 Table — Regression coefficients of linear regression models are given. Models included psychosis liability (high vs. low), childhood trauma (yes/no) and psychosis liability x childhood trauma as predictors and were corrected for sex, age, BMI, smoking, cannabis use, education, oral contraceptive use and psychotropic medication use. (DOCX) [file pone.0219139.s003.docx]

|  | Psychosis liability | | | |  | Childhood trauma | | | |  | Psychosis liability x childhood trauma | | | |
| --- | --- | --- | --- | --- | --- | --- | --- | --- | --- | --- | --- | --- | --- | --- |
|  | B | [CI] |  | p |  | B | [CI] |  | p |  | B | [CI] |  | p |
| BDNF | 0.06 | [-0.13; | 0.26] | 0.52 |  | -0.02 | [-0.23; | 0.19] | 0.87 |  | 0.01 | [-0.26; | 0.27] | 0.96 |
| CCL-2 | -0.28 | [-0.57; | 0.02] | 0.06 |  | 0.01 | [-0.30; | 0.31] | 0.97 |  | 0.10 | [-0.30; | 0.50] | 0.61 |
| CRP | -0.10 | [-0.88; | 0.67] | 0.79 |  | 0.02 | [-0.81; | 0.85] | 0.96 |  | 0.15 | [-0.91; | 1.20] | 0.78 |
| IFN-γ | 0.08 | [-0.21; | 0.38] | 0.57 |  | 0.20 | [-0.11; | 0.52] | 0.19 |  | -0.27 | [-0.67; | 0.13] | 0.18 |
| IGFBP-2 | 0.49 | [-0.34; | 1.33] | 0.25 |  | -0.23 | [-1.13; | 0.66] | 0.61 |  | -0.39 | [-1.53; | 0.75] | 0.50 |
| IL-6 | -0.12 | [-1.86; | 1.62] | 0.89 |  | -1.14 | [-3.00; | 0.72] | 0.23 |  | 1.19 | [-1.18; | 3.56] | 0.32 |
| PDGF | 0.26 | [-0.04; | 0.55] | 0.08 |  | 0.20 | [-0.11; | 0.51] | 0.21 |  | -0.41 | [-0.81; | -0.01] | 0.04 |
| SCF | -0.01 | [-0.28; | 0.26] | 0.93 |  | 0.09 | [-0.20; | 0.37] | 0.54 |  | -0.03 | [-0.40; | 0.33] | 0.86 |
| TNF-α | 0.42 | [-0.60; | 1.45] | 0.41 |  | -0.75 | [-1.85; | 0.36] | 0.18 |  | 0.72 | [-0.68; | 2.13] | 0.31 |
